# Identification of VWF as a Novel Biomarker in Lung Adenocarcinoma by Comprehensive Analysis

**DOI:** 10.3389/fonc.2021.639600

**Published:** 2021-04-22

**Authors:** Yi He, Ruijie Liu, Mei Yang, Wu Bi, Liuyin Zhou, Sai Zhang, Jin Jin, Xujun Liang, Pengfei Zhang

**Affiliations:** ^1^ NHC Key Laboratory of Cancer Proteomics, Department of Oncology, Xiangya Hospital, Central South University, Changsha, China; ^2^ National Clinical Research Center for Geriatric Disorders, Xiangya Hospital, Central South University, Changsha, China

**Keywords:** lung adenocarcinoma, differential expression genes analysis, co-expression analysis, VWF, bioinformatics

## Abstract

Lung adenocarcinoma (LUAD) is one of the most malignant tumors with high morbidity and mortality worldwide due to the lack of reliable methods for early diagnosis and effective treatment. It’s imperative to study the mechanism of its development and explore new biomarkers for early detection of LUAD. In this study, the Gene Expression Omnibus (GEO) dataset GSE43458 and The Cancer Genome Atlas (TCGA) were used to explore the differential co-expressed genes between LUAD and normal samples. Three hundred sixity-six co-expressed genes were identified by differential gene expression analysis and Weighted Gene Co-expression Network Analysis (WGCNA) method. Those genes were mainly enriched in ameboidal-type cell migration (biological process), collagen-containing extracellular matrix (cell component), and extracellular matrix structure constituent (molecular function). The protein-protein network (PPI) was constructed and 10 hub genes were identified, including IL6, VWF, CDH5, PECAM1, EDN1, BDNF, CAV1, SPP1, TEK, and SELE. The expression level of hub genes was validated in the GEPIA database, compared with normal tissues, VWF is lowly expressed and SPP1 is upregulated in LUAD tissues. The survival analysis showed increased expression of SPP1 indicated unfavorable prognosis whereas high expression of VWF suggested favorable prognosis in LUAD (p < 0.05). Based on the immune infiltration analysis, the relationship between SPP1 and VWF expression and macrophage, neutrophil, and dendritic cell infiltration was weak in LUAD. Quantitative real-time PCR (qRT-PCR) and western blotting were used to validate the expression of VWF and SPP1 in normal human bronchial epithelial (HBE) cell and three LUAD cell lines, H1299, H1975, and A549. Immunohistochemistry (IHC) was further performed to detect the expression of VWF in 10 cases LUAD samples and matched normal tissues. In summary, the data suggest that VWF is a potential novel biomarker for prognosis of LUAD.

## Introduction

Lung cancer is the leading cause of cancer death around the world (1). Lung adenocarcinoma (LUAD) is the common histological type of lung cancer, and LUAD comprising 40% of all lung cancer cases is the most common type of lung cancer. Despite advances in early diagnosis and treatment methods, the 5-year overall survival rate of LUAD patients remains low (2). Therefore, it’s imperative to discover new biomarkers for early diagnosis and treatment to improve the prognosis of patients with lung cancer.

Gene expression profiling based on high-throughput techniques has become a powerful tool to identify significant genes associated with LUAD progression. A study combining The Cancer Genome Atlas (TCGA) dataset and the Gene Expression Omnibus (GEO) dataset identified CX3CL1 overexpression as a positive prognostic factor in patients with LUAD (3). By regulating the cell cycle signaling pathway, KIF2C was identified as involved in the invasion and prognosis in LUAD (4). Recently, PKMYT1 was verified as a promising target to improve the radiosensitivity of LUAD (5). Weighted Gene Co-Expression Network Analysis (WGCNA) is an efficient method to explore the relationship between genes and phenotypes (6). By detecting the interested module of clinical trait and co-expressed module of related genes, WGCNA can help us to mine the key genes in cancer (7, 8) and predict the function of target genes, which identifies potential biomarker genes or therapeutic target (9, 10).

**Figure 1:**
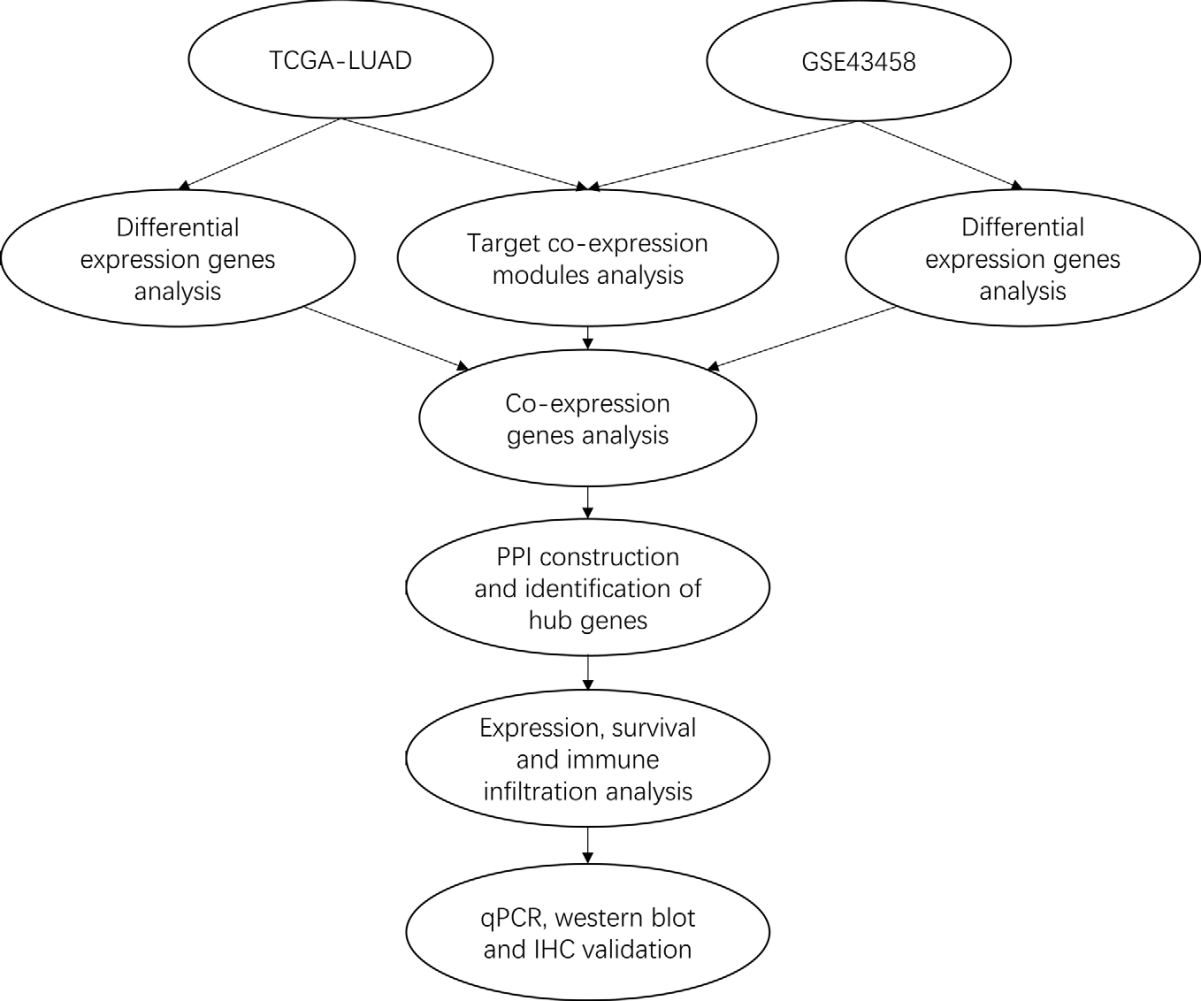
The workflow of the study.

In this study, we performed the integrated analysis by combining gene expression profiling from TCGA and GEO datasets with the WGCNA method to gain differential co-expression genes. Next, Gene Ontology (GO) and Kyoto Encyclopedia of Genes (KEGG) analysis of these genes were performed by clusterProlifer package. After that, protein-protein interaction (PPI) was constructed by STRING, survival analysis was performed with Survival package, immune infiltration analysis was used by TIMER. Then, qRT-PCR and western blotting were used to validate the expression of VWF and SPP1 in HBE cell and three LUAD cell lines. Finally, IHC was further detected the expression of VWF in 10 cases LUAD samples and matched normal tissues. Our results suggest that VWF could be a potential novel biomarker for prognosis of human LUAD. The workflow diagram is shown in **Figure 1**.

## Materials and Methods

### Datasets Source and Data Preprocessing

The RNAseq data and the gene expression profile of GSE43458 were downloaded from TCGA and GEO. Each dataset was processed using the R software (version 3.6.3, https://www.r–project.org/). TGCA-LUAD consists of 535 LUAD samples and 59 normal tissues, and RNAseq count data corresponding to 19,600 genes. The edgeR package (11) was used to screen differential expression genes, we selected the genes with a cpm (count per million) ≥1 for further study. Rpkm function was used to filter the data, which is calculated by dividing gene counts by gene length, 15,127 genes with rpkm values were picked for the next study. GSE43458 contains 80 lung adenocarcinoma samples and 30 normal lung tissues. The matrix files of GSE43458 were downloaded by using the GEOquery (12) package and the AnnoProbe package was used to convert the gene probe IDs to the gene symbols. After that, the limma package (13)was used to identify DEGs. As a result, 18,821 genes were selected for further analysis.

### Identification of Key Co-expression Modules Using WGCNA

We constructed gene co-expression networks by the WGCNA package based on the gene expression profile of TGCA-LUAD and GSE43458. The genes with a standard deviation >0.5 were set the criteria. WGCNA was used to explore the relationship between genes and sample traits by grouping highly functionally related genes into modules. Firstly, soft powers β = 6 and 12 were selected using the pick SoftThreshold function to obtain a scale-free network. Next, the adjacency matrix was built according to the Pearson correlation of all gene pairs and transformed into the topological overlap matrix (TOM) as well as the corresponding dissimilarity (1-TOM). Then, performing hierarchical clustering dendrogram on the 1-TOM matrix to divide similar gene expression into different gene co-expression modules. DynamicTreeCut algorithm was used to conduct hierarchical clustering about similar expression genes and classify them into the same gene modules. The trait-modules relationship was estimated by the Pearson correlation coefficient, the module with a high value was selected for subsequent study.

### Screening for Interest Differential Expression Genes

An adjusted p-value <0.05 and the absolute of log2 fold change (log2FC) >1 were set as the cut-off criteria. The DEGs of TCGA-LUAD and GSE43458 datasets were presented in the volcano plot using the ggplot2 package. FunRich software (14), a functional enrichment and interaction network analysis tool, was used to screen the overlapping genes among the TCGA-LUAD, GSE43458, and the two co-expression genes that came from the co-expression network. These genes were considered to be of prognostic value.

### Functional Enrichment Analysis

The clusterProlifer package (15) was used to perform Gene Ontology (GO) (16) analysis and Kyoto Encyclopedia of Genes and Genomes (KEGG) pathway enrichment analysis, which included biological processes, cellular components, molecular functions. Adjusted p-value <0.05 was set as the threshold value.

### PPI Network Construction

The Search Tool for the Retrieval of Interacting Genes(STRING, http://string-db.org) (17) was used to construct the interactive network of the overlapping DEGs and subsequently was visualized using the Cytoscape (18) software. The combined score <0.4 was set as the cut-off criteria. In this study, we used the plugin CytoHubba (19) and MCODE to identify the hub genes and functional modules in the PPI network.

### Expression Level and Prognostic Value of Hub Genes

GEPIA (20) (http://gepia.cancerpku.cn/index.html) was used to study the gene expression of normal group and tumor groups based on different clinical stages. The Survival package was used to dig out the relationship between the overall survival (OS) and the hub genes based on TCGA data. We divided the hub genes into two groups based on their median expression value. The gene with the Log-rank test p < 0.05 was considered statistically significant. We also performed analyzed the correlation of gene expression and clinical parameters in LUAD. The immune infiltration analysis of hub genes was performed by TIMER (21) (https://cistrome.shinyapps.io/timer/).

### Cell Culture

The LUAD cell lines (H1299, H1975, and A549) were purchased from Chinese Academy of Sciences Cell Bank (Shanghai, China) and HBE cell was maintained in our laboratory. HBE served as control. All cells cultured in RPML-1640 medium (Gibco, USA) with 10% fetal bovine serum (Biowest S.A.S, France) and 1% penicillin-streptomycin (Gibco, USA) in a humidified incubator at 37°C containing 5% CO_2_.

### RT-PCR

The TRIzol method was used to extract total RNA from cell lines, then transformed into cDNA by using SureScriptTM First-Strand cDNA Synthesis Kit (GeneCopoeia, Guangzhou, China). The expression level of SPP1 and VWF were measured by qRT-PCR using the commercial kit (BlazaTaqTM SYBR^®^ Green qPCR Mix 2.0 (GeneCopoeia, Guangzhou, China) and a LightCycler 480 II System 373 (Roche, USA). β-actin set as the internal control. The primer sequences are list follows SPP1-F: AGCAGAATCTCCTAGCCCCA, SPP1-R: TGGTCATGGCTTTCGTTGGA; VWF-F: TTGTGGGAGATGTTTGCCTAC, VWF–R: CCTCTCTCATTGACCTTGCAG; ACTIN-F: CTTCGCGGGCGACGAT, ACTIN-R: CCATAGGAATCCTTCTGACC. The expression quantification was obtained with the 2^−ΔΔCt^ method.

### Western Blotting

Cells were lysed in IP lysis buffer containing protease inhibitors and then measured protein concentration by BCA assay. An equal amount of protein from cells loaded into the 8% SDS-PAGE, transferred to polyvinylidene difluoride membranes (PVDF), then incubated with primary antibody against VWF (1:500, Cell Signaling Technology, Shanghai, China) and SPP1 (1:2,000, Proteintech, Wuhan, China) at 4°C overnight, followed by incubation with peroxidase-conjugated secondary antibody (1:5,000, Abbkine, Wuhan, China) at room temperature for 1 h, finally visualized by enhanced chemiluminescence detection. The β-actin (1:5,000, Abbkine, Wuhan, China) was used as internal references in this study.

### Immunohistochemistry and Evaluation of Staining

Ten cases of fresh-frozen and paraffin-embedded LUAD tissues and adjacent non-cancerous tissues of LUAD patients were collected from the Department of Thoracic Surgery, Xiangya Hospital of Central South University, China. The research was approved by the local ethics committee (No. 202103046). The tissue was deparaffinized, rehydrated, and treated with an antigen retrieval in 0.01M citrate salt buffer (ZSGB-BIO, Beijing, China). After incubation of anti-VWF rabbit antibody (1:400, Abcam, Shanghai, China) overnight at 4°C, the tissue sections were incubated with horseradish peroxidase-conjugated anti-rabbit antibody (1:1,000, ZSGB-BIO, Beijing, China). Finally, the sections were stained with DAB+ substrate-chromogen solution (ZSGB-BIO, Beijing, China) and counterstained with hematoxylin. In negative controls, primary antibodies were omitted.

Two investigators assessed the immunostained result in a blinded fashion. The expression level of VWF was evaluated by intensity and percentage of positive cells according to a semi-quantitative method (22). The intensity was defined as 0 for negative staining, 1 for weak staining, 2 for moderate staining, and 3 for strong staining. Percentage of positive cells was quantified as 0 for ≤5% positive cells, 1 for 6–25%, 2 for 26–50%, 3 for 51–75%, and 4 for ≥76%. The score was calculated by multiplying the score of staining intensity and percentage of positive cells.

### Statistical Analysis

Statistical analyses were performed using GraphPad Prism 8 software. The result of RT-PCR was compared using the Students’ t-test. Wilcoxon test was used to estimate the relationship between gene expression and LUAD patients’ clinical parameters. The difference of VWF in LUAD tissue and normal lung tissues was analyzed by Mann–Whitney U-test. The p-value <0.05 was considered statistically significant.

## Results

### Construction of Weighted Gene Co-expression Modules

**Figure 2:**
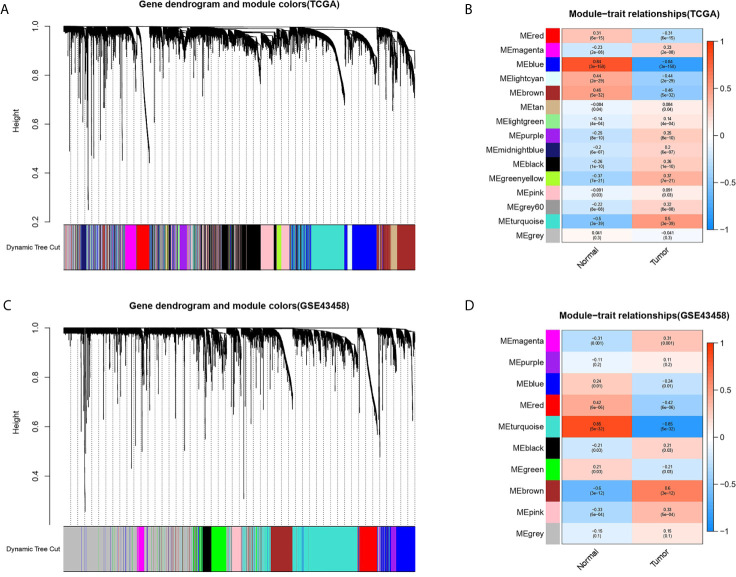
Identification of modules associated with clinical trait based on TCGA and GEO database. **(A, C)** The cluster dendrogram of genes in different modules based on 1-TOM matrix in TCGA and GSE43458 platform. **(B, D)** Heatmap of correlation between modules and clinical traits such as tumor and normal in TCGA and GSE43458 platform. Each row and column represent the module and the relationship between module and clinical traits (tumor and normal), which showed by p-value.

The gene co-expression network was constructed from TCGA-LUAD and GSE43458 dataset using the WGCNA package. There were 14 and 9 modules identified in the TCGA-LUAD (**Figure 2A**) and the GSE43458 (**Figure 2C**). Then, the module-trait relationship was shown in the heatmap to estimate the relevance between each module and two clinical traits (tumor and normal). As we can see from **Figures 2B, D**, the blue module (r = 0.84, p = 3e-158) in TCGA-LUAD and the turquoise module (r = 0.85, p = 5e-32) in GEO43458 had the strongest correlation with normal tissues. The genes in the TCGA blue module were mainly enriched in positive regulation of transcription from RNA polymerase II promoter (biological process), calcium ion binding (molecular function), and integral component of membrane (cell component). The genes in the GEO turquoise module were mainly enriched in cell adhesion (biological process), calcium ion binding (molecular function), and plasma membrane (cell component).

### Screening for Interest Differential Expression Genes

**Figure 3:**
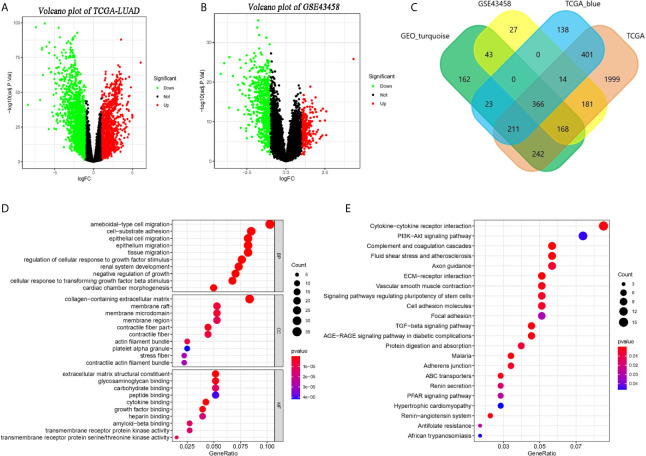
Identification of co-expressed genes and related functional enrichment analysis. **(A, B)** The volcano map of differential expression genes (DEGs) in TCGA and GSE43458 platform. The cutoff is |logFC| > 1 and adj.p < 0.05. **(C)** The Venn diagram of genes among four DEGs list. Three hundred sixty-six co-expressed genes were found in two platform and two co-expression modules. **(D, E)** The Gene Ontology (GO) and KEGG analysis of co-expressed genes.

Setting the standard that adjusted p < 0.05 and the absolute of logFC larger than 1, we identified 3,582 (**Figure 3A**) and 799 (**Figure 3B**) DEGs in TCGA-LUAD and GSE32665. And 1,153 genes and 1,215 genes were identified in the TCGA-blue module and GEO- turquoise module. In addition, 366 overlapping genes were found by the Veen diagram (**Figure 3C**).

### GO and KEGG Pathway Enrichment Analyses of Overlapping Genes

The GO and KEGG pathway analysis of 366 overlapping genes were processed by clusterProlifer package. In biological process (BP) term, overlapping genes mainly in ameboidal-type cell migration, cell-substrate adhesion, and epithelial cell proliferation. As of cell component (CC) term, overlapping genes were extremely involved in the collagen-containing extracellular matrix, membrane raft, and membrane microdomain. In molecular function (MF) term, 366 genes were mainly associated with extracellular matrix structure constituent, glycosaminoglycan binding, and carbohydrate-binding (**Figure 3D**). KEGG analysis indicated those genes were mainly involved in cytokine-cytokine receptor interaction, PI3K-Akt signaling pathway, and complement and coagulation cascades (**Figure 3E**).

### Protein-Protein Interaction (PPI) Network Construction and Screening of Hub Genes

**Figure 4:**
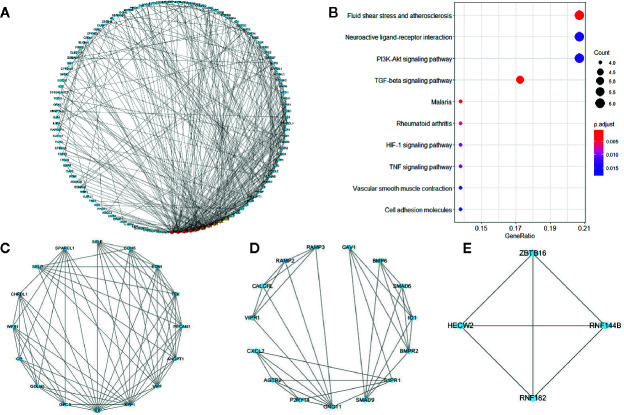
Pro****tein-protein interaction network of co-expressed genes and modules analysis. **(A)** PPI structure of co-expressed genes. **(B)** KEGG pathway analysis of genes in the top three modules. **(C–E)** Top three modules identified by MCODE plugin in Cytoscape.

The PPI network of overlapping genes consisted of 365 nodes with 806 edges, and the average clustering coefficient was 0.367 (**Figure 4A**). The top 10 hub genes were screened by the CytoHubba according to their degree values, including IL6, VWF, CDH5, PECAM1, EDN1, BDNF, CAV1, SPP1, TEK, and SELE. A total of 17 modules were identified *via* the MCODE plugin and the top 3 modules are shown in (**Figures 4C–E**). KEGG pathway analysis showed that hub genes in the three modules were mainly associated with fluid shear stress and atherosclerosis, neuroactive ligand-receptor interaction, and PI3K-Akt signaling pathway (**Figure 4B**).

### Expression Level and Prognostic Value of Hub Genes

**Figure 5:**
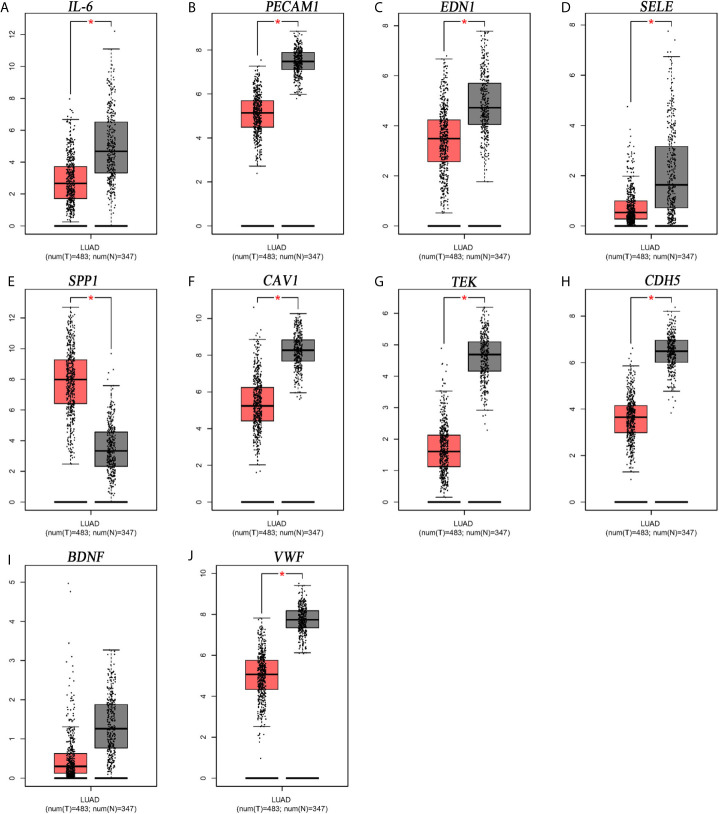
Expression level analysis of hub gens. **(A)** IL6; **(B)** PECAM1; **(C)** EDN1; **(D)** SELE; **(E)** SPP1; **(F)** CAV1; **(G)** TEK; **(H)** CDH5; **(I)** BDNF; **(J)** VWF. *p<0.05.

**Figure 6:**
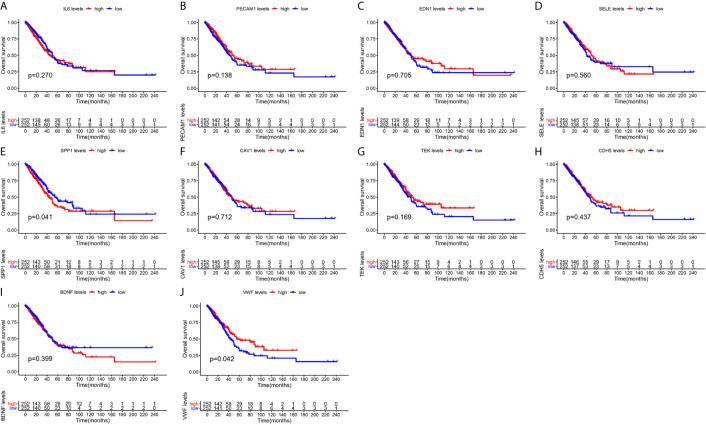
Survival analysis of hub gens. **(A)** IL6; **(B)** PECAM1; **(C)** EDN1; **(D)** SELE; **(E)** SPP1; **(F)** CAV1; **(G)** TEK; **(H)** CDH5; **(I)** BDNF; **(J)** VWF.

**Figure 7:**
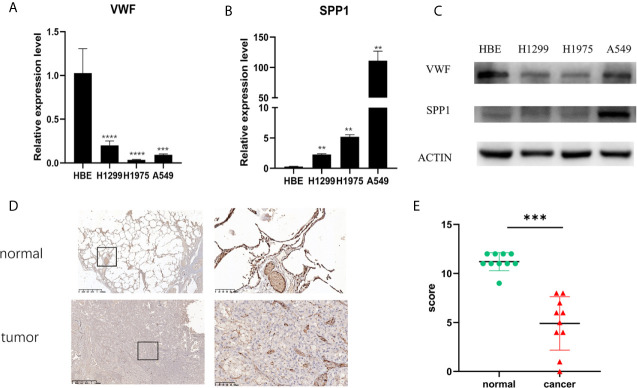
Validation of mRNA and protein expression level. The mRNA expression level of VWF **(A)** and SPP1 **(B)** in HBE, H1299, H1975, and A549 cell lines. **p<0.01,***p<0.001,****p<0.0001. And protein expression of VWF and SPP1 validated using western blotting **(C)**. Each experiment was carried out three times. A representative immunohistochemistry image of VWF in LUAD and adjacent non-tumor tissues. Scale bar = 100 µm **(D)**. The scatter plot of immunohistochemistry score of LUAD and matched normal tissues. **(E)**. ***p< 0.001.

From **Figure 5**, it can be seen that 9 of the 10 hub genes were lower expressed in lung adenocarcinoma compared with normal tissues, whereas expression of SPP1 was relatively higher expressed in the LUAD tissues. The OS analysis was performed using the survival package. As shown in **Figure 6**, the high expression of VWF was concerned with favorable prognosis, and the high expression of SPP1 was associated with worse prognosis (p < 0.05). Next, we performed RT-PCR, western blotting, and immunohistochemistry experiments to validate the mRNA and protein levels of SPP1 and VWF in LUAD cell lines and tissues. The result of qRT-PCR (**Figures 7A, B**) showed a higher level of SPP1 and a lower level of VWF in three LUAD cell lines compared with normal control (HBE). However, the western blotting analysis indicated compared with the low expression of VWF in three LUAD cell lines, SPP1 was highly expressed in only one cell line (A549), not in H1299 and H1975 cell lines (**Figure 7C**). As for tissue samples, Li group had validated the difference of SPP1 expression in LUAD patients using IHC (23), we just performed an analysis of VWF expression in IHC, the IHC analysis presented low expression level in LUAD tissue compared with normal lung tissue (**Figures 7D, E**).

**Figure 8:**
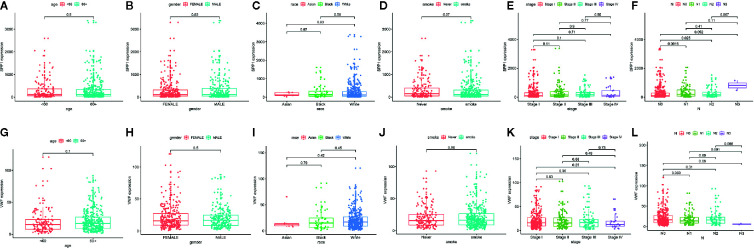
The Correlation between hub gene expression and clinical parameters in lung adenocarcinoma. Association between SPP1 expression and age **(A)**, gender **(B)**, race **(C)**, smoking habit **(D)**, stage **(E)**, and N status **(F)**. Association between VWF expression and age **(G)**, gender **(H)**, race **(I)**, smoking habit **(J)**, stage **(K)**, and N status **(L)**.

**Figure 9:**
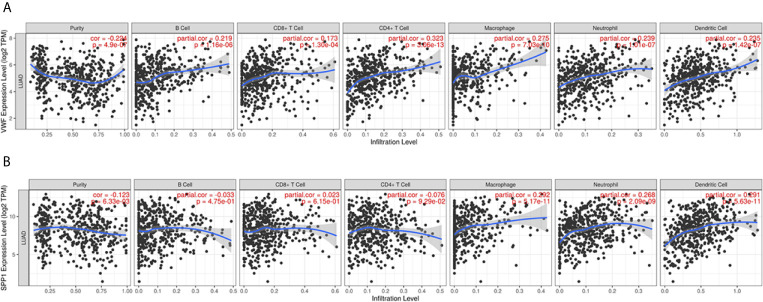
Immune infiltration analysis of Hub genes. The relationship between VWF **(A)** and SPP1 **(B)** and infiltration of immune cells including purity, B cell, CD4+T cell, CD8+T cell, and so on.

We also extracted data from TCGA-LUAD and GSE43458 dataset to explore the relationship between hub gene expression and clinicopathological parameters in LUAD, the VWF and SPP1 expression were not significantly associated with gender, age, race, smoking habit, and stage (**Figure 8**). Furthermore, to dig out the function of hub genes in the immune microenvironment, we used the TIMER dataset to estimate the correlations of two hub genes with tumor purity and infiltrating immune cells (**Figure 9**). There was a weak relationship between VWF, SPP1 expression, and infiltration of immune cells in LUAD (**Figures 9A, B**). VWF expression was associated with the infiltration level of B cell (r = 0.219, p = 1.16e-06), CD8+T cell (r = 0.173, p-1.30e-04), macrophage (r = 0.275, p = 7.03e-10), neutrophil (r = 0.239, p = 1.01e-07), and dendritic cell (r = 0.235, p = 1.43e-07) in LUAD. SPP1 expression level in LUAD was correlated with infiltrating CD4+T cell (r = −0.076, p = 9.39e-02), macrophage (r = 0.292, p = 5.17e-11), neutrophil (r = 0.268, p = 2.09e-09), and dendritic cell (r = 0.291, p = 5.63e-11).

## Discussion

In recent decades, lung adenocarcinoma has become one of the most common malignancies in lung cancer. Most patients are diagnosed in the late stage because the early symptoms are not obvious. Through the treatment and diagnostic approaches have been improved, the mortality and incidence of lung adenocarcinoma remain high. Therefore, there is an urgent need to discover potential biomarkers for the prediction of the development and prognosis of LUAD.

In this study, WGCNA was used to construct the co-expression network of TCGA-LUAD and GSE43458 databases and identify relative gene modules. Three hundred sixty-six overlapping genes were found using integrated bioinformatics analysis. The overlapping genes were mainly involved in ameboidal-type cell migration (BP), collagen-containing extracellular matrix (CC), and extracellular matrix structural constituent (MF). Also, the plugin in Cytoscape, CytoHubba, and MCODE, were used to identify the hub genes and screen modules in the PPI network. The hub genes were IL6, VWF, CDH5, PECAM1, EDN1, BDNF, CAV1, SPP1, TEK, and SELE. Compared with normal tissues, only the SPP1 expression increased in LUAD tissues, while the rest hub genes decreased. The survival analysis indicated high expression of SPP1 was associated with poor overall survival in LUAD, while low expression of VWF was related to favorable overall survival. The protein expression level of SPP1 in H1299 and H1975 cell lines was inconsistent with mRNA level, which may be disturbed by post-translation, such as phosphorylation and ubiquitin.

Our workflow of the study was similar to the work (24). The other work screened the DEGs using differential gene expression analysis and identified hub genes in the PPI network, then performed related analysis to explore the relationship of hub genes and LUAD. The hub genes expression validated in RT-PCR and western blotting. CDH5, PECAM1, and VWF may play an important role in LUAD. We screened the co-expression genes using differential gene expression and WGCNA, identified hub genes from the PPI network, following the survival, and immune infiltration analysis, then expression validation using RT-PCR, western blotting, and IHC. VWF and SPP1 can be used for further study. We all found the hub genes (IL6, VWF, CDH5, PECAM1, SPP1) from different PPI networks, and VWF and SPP1 expression were associated with LUAD patients’ prognosis. But some differences need to be noticed. Firstly, the selection of data sources from the different platforms is more representative. Secondly, the idea of the study was different. WGCNA was powerful to find trait-related genes. Thirdly, immune infiltration analysis and many validation experiments have further enhanced the importance of hub genes. The same hub genes identified from different methods may play a role in the development of LUAD. SPP1, secreted phosphoprotein 1, also named osteopontin (OPN), is a secreted calcium-binding phosphorylation protein. SPP1 plays an important role in the regulation of biological processes such as cell proliferation, angiogenesis, inflammation, and apoptosis (25, 26). More evidence shows that SPP1 was overexpressed in various types of cancers and contributes to the occurrence and development of tumors, including colorectal cancer, cervical cancer, gastric cancer, and so on (27–30). The result of our finding that SPP1 may be a potential biomarker for lung adenocarcinoma was consistent with several studies (31, 32). More reports had illustrated the relationship between the expression level of SPP1 and lung adenocarcinoma. For example, by inhibiting autophagy and apoptosis, SPP1 promotes the development of small cell lung cancer (33). SPP1 enhanced the second-generation epidermal growth factor receptor (EGFR) tyrosine kinase inhibitor (TKI) resistance in lung cancer, which offered a new scheme for targeted therapy that inhibiting SPP1 might be a therapeutic target to overcome afatinib resistance (34). Meanwhile, SPP1 mediating macrophage polarization leads to upregulation of PD-L1 and facilitates immune escape in lung adenocarcinoma, which also suggests a potential therapeutic target for lung cancer (35). These studies further presented the importance of SPP1 in the mechanism of metastasis and invasion of the tumor.

VWF, von Willebrand factor, a multimeric protein produced by endothelial cells, platelets, and megakaryocytes, dedicates to primary hemostasis, mediating platelets adhesion and acting as a carrier protein for coagulation factor (F) VIII (36, 37). The VWF is highly expressed in cancers such as kidney renal clear cell carcinoma, glioblastoma multiforme, and liver hepatocellular carcinoma, while lowly expressed in tumors including colon adenocarcinoma, breast invasive carcinoma, and uterine corpus endometrial carcinoma. Due to the significance of hemostasis, VWF plays a role in cancers by inhibiting angiogenesis and apoptosis (38–40). The high expression of VWF relates to endothelial damage and is used for indicating many pathological situations, including atherosclerosis, cardiovascular diseases, and cancers (41–44). Besides, scientists pay attention to explore the relationship between the expression level of VWF and lung cancer. Liu group found that VWF expression in endothelial cells of LUAD is elevated by the cancer cell-derived secretome through GATA3-mediated transcription (45); According to a study, which digs out the association between VWF and its cleaving protease (ADAMTS-13) in lung cancer metastasis, the elevation of VWF/ADAMTS-13 ratio may serve as an independent predictive factor for mortality in patients with advanced NSCLC (46). Another useful study considered the interference of different blood groups while studying the expression levels of VWF and ADAMTS-13 in lung cancer patients, showed that increased VWF and decreased ADAMTS-13 promote the invasion of lung cancer and non-O blood group is a risk factor for increased VWF and FVIII in plasma (47). The special molecular mechanism of how VWF affects lung adenocarcinoma is still underway. Thus, further studies are needed to confirm this contention.

Although we have preliminary screened the potential prognostic biomarkers of LUAD, there still exist some limitations. On one hand, the number of samples is insufficient to avoid bias, on the other hand, more in-depth studies are needed in the future. Our analysis can provide valuable information for researchers to find possible genes and pathways concerned with LUAD for better understanding the molecular mechanism of LUAD carcinogenesis.

In summary, we identified 10 hub genes by integrating the GEO and TCGA dataset with WGCNA, and further showed that VWF down-regulation might be a prognostic biomarker in LUAD. These findings reported here could have clinical value in prognosis of LUAD, and provide valuable information for further studying the roles of these differential hub genes in LUAD.

## Data Availability Statement

Publicly available datasets were analyzed in this study. This data can be found here: https://portal.gdc.cancer.gov/ and https://www.ncbi.nlm.nih.gov/gds.

## Ethics Statement

The studies involving human participants were reviewed and approved by the ethics committee of Xiangya Hospital of Central South University. The patients/participants provided their written informed consent to participate in this study.

## Author Contributions

LZ, SZ, and WB: design and methodology. XL and YH: software and data procession. JJ and YH: validation. YH, RL, MY, and WB: writing-original draft preparation. XL and PZ: writing-review, editing, and supervision. All authors contributed to the article and approved the submitted version.

## Funding

This work was supported by the National Natural Science Foundation of China (Nos. 82073008), Key Research and Development Project of Hunan Province (2020SK2071), and Natural Science Foundation of Hunan Province (2020JJ4924).

## Conflict of Interest

The authors declare that the research was conducted in the absence of any commercial or financial relationships that could be construed as a potential conflict of interest.
