# Supplementary figures and images for: Identification of VWF as a Novel Biomarker in Lung Adenocarcinoma by Comprehensive Analysis
Source: Front Oncol. 2021 Apr 22;11:639600. doi: 10.3389/fonc.2021.639600 (PMC8100660; doi:10.3389/fonc.2021.639600)

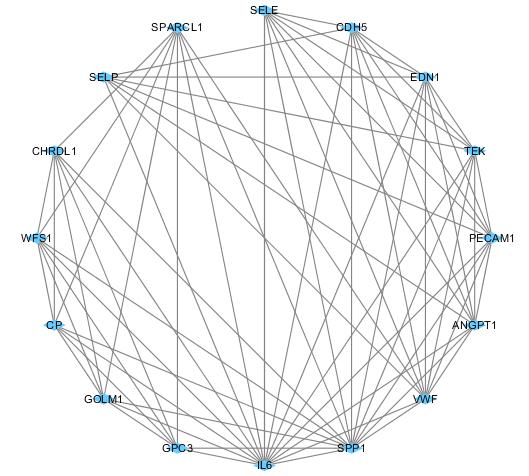

Supplement: Supplementary file 1 [file Image_1.jpeg]

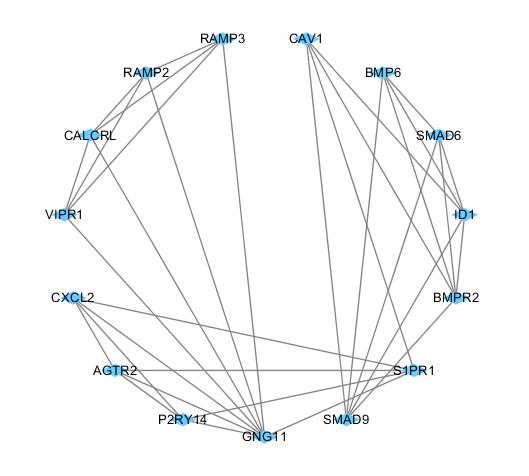

Supplement: Supplementary file 2 [file Image_2.jpeg]

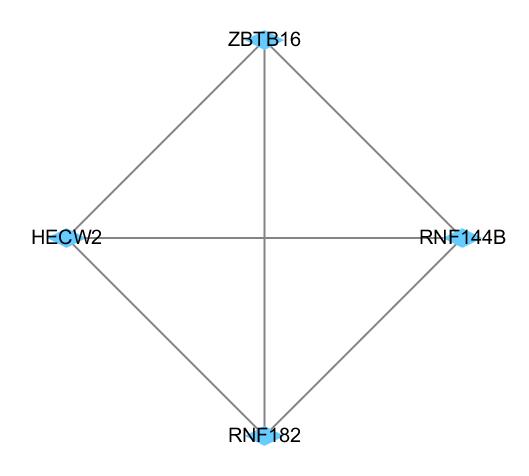

Supplement: Supplementary file 3 [file Image_3.jpeg]

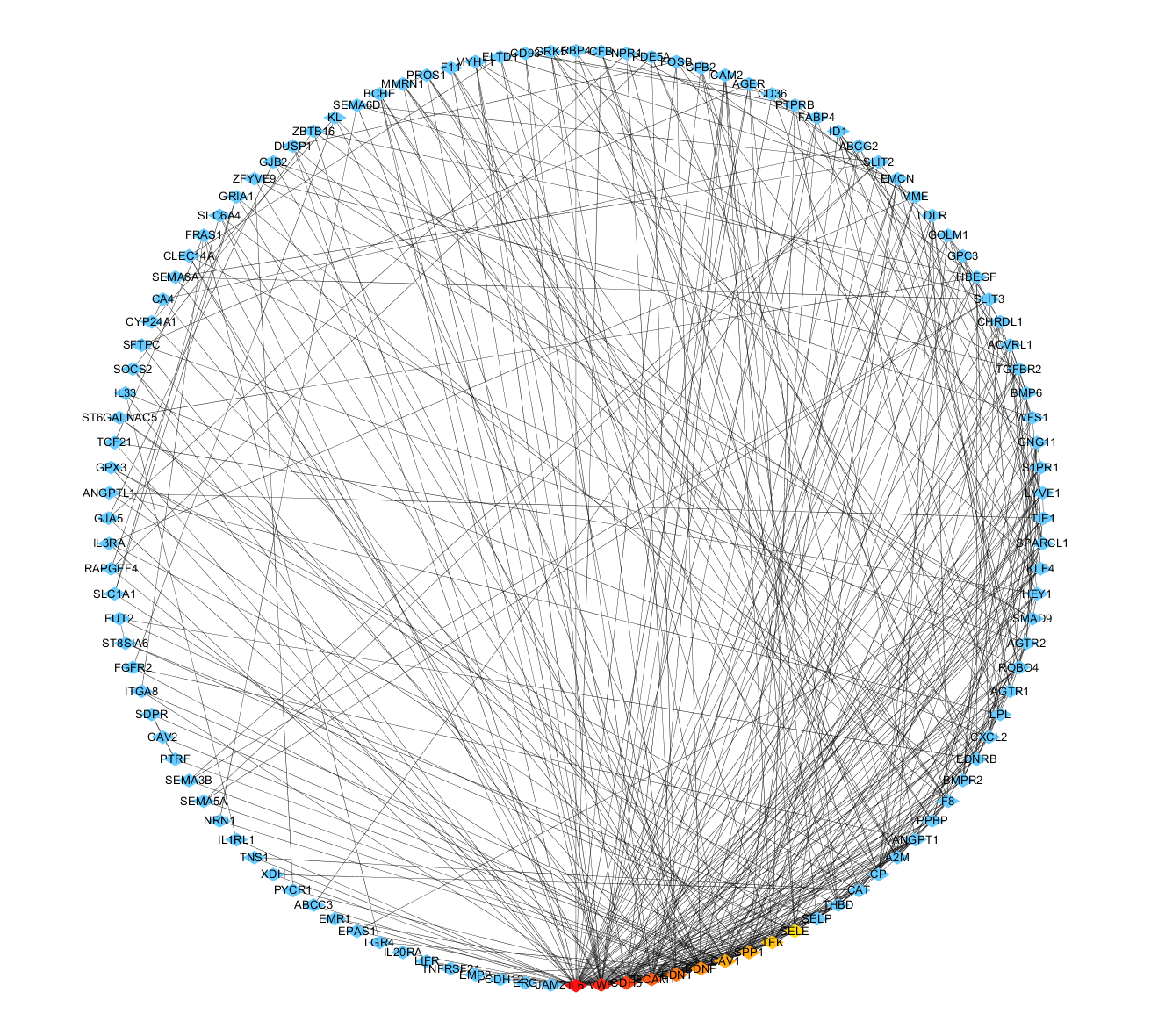

Supplement: Supplementary file 4 [file Image_4.jpeg]
